# Change in choroidal blood flow and choroidal morphology due to segmental scleral buckling in eyes with rhegmatogenous retinal detachment

**DOI:** 10.1038/s41598-017-05126-1

**Published:** 2017-07-20

**Authors:** Takeshi Iwase, Misato Kobayashi, Kentaro Yamamoto, Kosei Yanagida, Eimei Ra, Hiroko Terasaki

**Affiliations:** Department of Ophthalmology, Nagoya University Graduate School of Medicine, Nagoya, Japan

## Abstract

Although scleral buckling is a well-established surgical treatment for rhegmatogenous retinal detachment (RRD), the procedure can reportedly cause problems in the ocular circulation. Segmental scleral bucking without a concomitant encircling procedure was performed on 46 eyes with successfully reattached macula-on RRD. Choroidal blood flow was assessed using laser speckle flowgraphy. Spectral-domain optical coherence tomography was used to image macular regions, to measure the subfoveal choroidal thickness (SFCT), and to calculate the luminal and the stromal areas by the binarization method preoperatively and 1, 4, 8 and 12 weeks postoperatively. Choroidal mean blur rate at the macula did not significantly change, while that at the buckle and unbuckle side significantly reduced at 8 weeks postoperatively in the operated eye (P = 0.007 and P = 0.017, respectively). The SFCT and the luminal area increased temporarily 1 week following surgery in the operated eye (P < 0.001). The trend of SFCT with time coincided with that of the luminal area (P < 0.001). Venous drainage obstruction induced by compression force of scleral buckling leads to SFCT thickening in the acute postoperative phase. The macular choroidal blood flow might be less susceptible because the blood flow at the macula, in contrast to the other areas, does not change following segmental scleral buckling.

## Introduction

Rhegmatogenous retinal detachment (RRD), which is the detachment of the sensory retina from the retinal pigment epithelium (RPE) caused by breaks in the retina, is a sight-threatening pathology^1^. RRD is usually treated with either scleral buckling alone or pars plana vitrectomy (PPV) with or without concomitant scleral buckling.

Although scleral buckling is a well-established surgical treatment for RRD and the anatomical success rate is high^2^, the procedure can reportedly cause problems in the ocular circulation^3–6^. Experimentally, it was found that encircling buckling significantly decreased retinal and choroidal circulation using the microsphere technique in rabbit eyes^3^. Several authors reported that scleral buckling procedures reduced retinal^4, 5, 7^ and choroidal blood flow^8–10^ due to compressional mechanisms^3, 11^. However, very few reports evaluated choroidal blood flow following segmental scleral buckling without the encircling procedure for eyes with RRD.

A variety of techniques have been developed for measuring retinal blood flow, including fluorescein angiography, radioactive microspheres, hydrogen clearance, the laser Doppler technique^4, 6^, colour Doppler ultrasonography^12^, and the pulsatile technique^8^. Time intensiveness or problems with reproducibility have, however, hampered their widespread use. Accordingly, the number of eyes is low in most of studies using these methods.

Laser speckle flowgraphy (LSFG) (Softcare Co., Ltd., Fukutsu, Japan) is a non-invasive, real-time method used to measure relative blood flow for 4 s without the administration of contrast agents^13, 14^. LSFG can detect the speckle contrast pattern produced by the interference of illuminating laser light scattered by the movement of erythrocytes in the blood vessels, and it enables the measurement of relative blood flow in the vessels, expressed as the mean blur rate (MBR)^13^. The measurements have excellent reproducibility: the coefficient of variation (COV) was 4.7 on choroidal blood flow^15^. Therefore, LSFG was considered suitable for measuring ocular –including choroidal–blood flow.

Kimura *et al*. reported a temporary thickening of subfoveal choroidal thickness (SFCT) using spectral domain optical coherence tomography (SD-OCT) after segmental scleral buckling surgery^16^. They speculate that haemostasis and inflammation caused the temporal increase in SFCT.

The choroid is composed mainly of vessels and stroma (extravascular tissue) lacking a well-organised structure. However, it is difficult to differentiate the luminal area from the stromal area in the choroid *in vivo*. Recently, Sonoda *et al*. reported the use of a binarization method involving SD-OCT images, which can differentiate the choroidal luminal area from the stromal area and quantify the areas using Image-J software with high repeatability^17^. Therefore, it is important to understand what variation in the choroidal structure affects SFCT and to compare choroidal with luminal area blood flow.

Thus, this study aimed to evaluate the change in choroidal blood flow using LSFG following segmental scleral buckling procedures and the relationship between choroidal blood flow change and morphological change using SD-OCT.

## Results

### Patient demographics

Between August 2013 and March 2015, 114 eyes of 114 patients with RRD underwent segmental scleral buckling at our department for the repair of RRD. Of those, 68 eyes were excluded for macula-off (n = 42), vitreous haemorrhage (n = 1), glaucoma (n = 3), diabetic retinopathy (n = 1), or an incapacity to attend regular follow-up visits (n = 21). Included in final analysis were 46 eyes with macula-on RRD (mean age, 44.6 ± 17.4 years). Patient demographics and surgical procedures are shown in Table 1. The number of eyes undergoing examinations in each observation period was 46 at 1 week, 36 at 4 weeks, 32 at 8 weeks and 31 at 12 weeks following surgery. There were no cases of scleral buckling straddling extraocular vortex veins. The extent of quadrant-wise buckle was 91.5 ± 21.6 degrees.

**Table 1:** Clinical characteristics of subjects.

Characteristics	mean ± SD
Age (years)	44.6 ± 17.4
Male/Female	28/18
Preoperative BCVA (Log MAR)	0.13 ± 0.37
Preoperative IOP (mmHg)	13.1 ± 3.3
Refraction (diopter)	−4.4 ± 3.3
Axial length (mm)	25.89 ± 1.71
Phakic eye/Psuedophakic lens	44/2
Extent of retinal detachment (%)	
1 quadrant	71.7
2 quadrants	23.9
3 quadrants	4.4
Number of retinal holes/tears (%)	
1	73.9
≧2	26.1
Extent of quadrant-wise buckle (degrees)	91.5 ± 21.6

BCVA: best-corrected visual acuity, LogMAR: logarithm of the minimal angle of resolution, IOP: intraocular pressure.

### Changes in choroidal MBR

**Figure 1:**
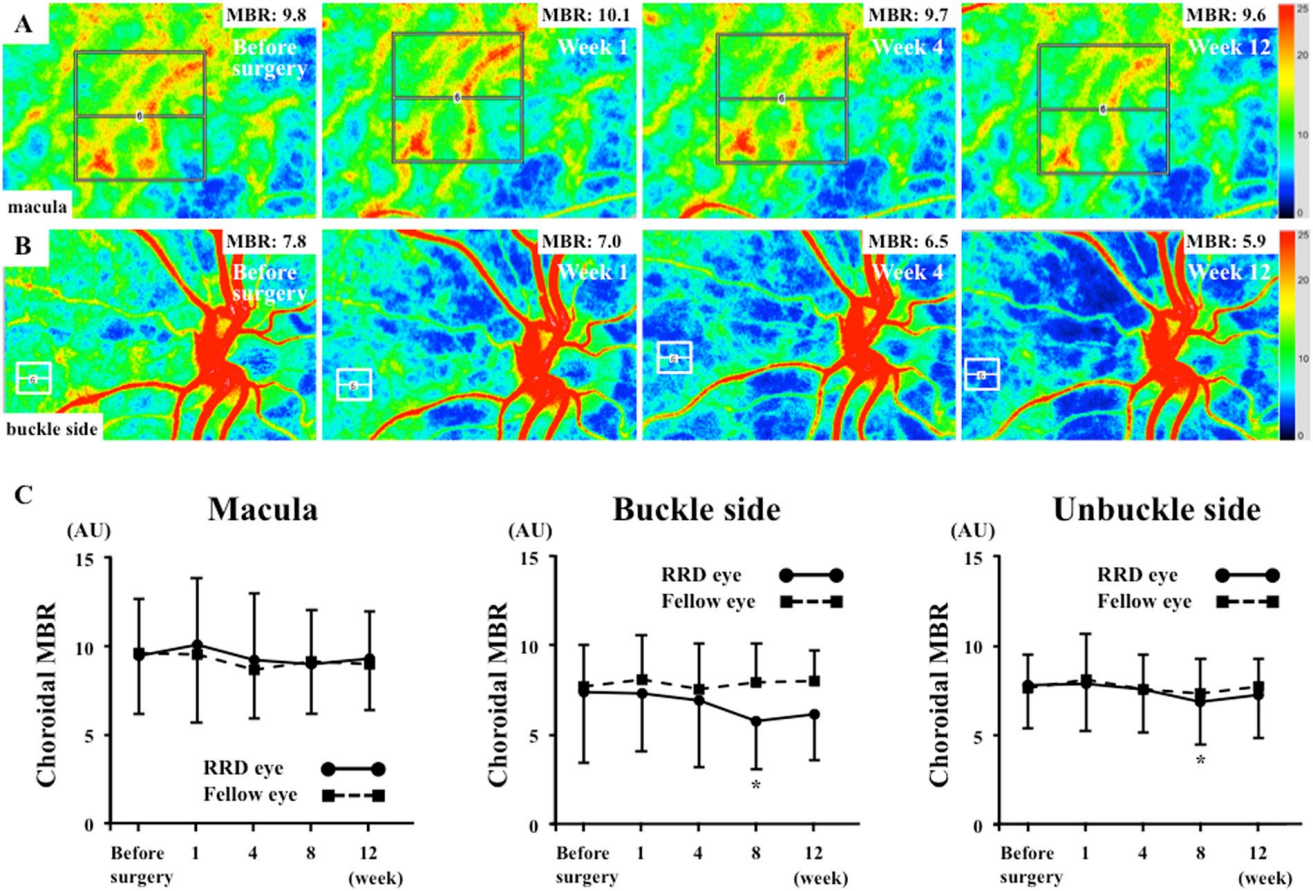
Representative composite colour maps using the mean blur rate (MBR) as measured by laser speckle flowgraphy (LSFG) in eyes with rhegmatogenous retinal detachment (RRD) preoperatively and 1, 4 and 12 weeks postoperatively at the macula (**A**) and the buckle side (**B**). The red colour indicates a high MBR, and the blue colour indicates a low MBR. To measure choroidal MBR, the centre of a square was set at the fovea (250 × 250 pixels, degree: 6.31° × 6.31°) and the buckle and unbuckle side (60 × 60 pixels, degree: 1.5° × 1.5°). The mean change in MBR in the operated eye and the fellow eye. There was no significant change in macular choroidal blood flow during follow-up in the operated eye group or the fellow eye group (**C**). There was a significant reduction in choroidal MBR at the buckle and unbuckle side at 8 weeks after surgery compared with before surgery in the affected eyes (P = 0.007, P = 0.017, respectively).

In eyes affected by RRD, the mean macular choroidal MBR was 9.4 ± 3.2 arbitrary units (AU) before surgery, 9.9 ± 3.7 AU at 1 week, 9.4 ± 3.7 AU at 4 weeks, 8.9 ± 3.1 AU at 8 weeks and 9.3 ± 2.6 AU at 12 weeks following surgery (Fig. 1). There was no significant change in macular choroidal MBR over time in the affected or unaffected opposite eyes (Table 2). There was no significant difference in macular choroidal MBR between the affected eye group and the unaffected opposite eye group in the entire observation period.

**Table 2:** Change in variable parameters with time.

	Parameter	Before surgery	Week 1	Week 4	Week 8	Week 12	*P*-value
RRD eye	MBR at fovea (AU)	9.4 ± 3.2	9.9 ± 3.7	9.4 ± 3.7	8.9 ± 3.1	9.3 ± 2.6	0.065
	MBR at buckle side (AU)	7.4 ± 4.0	7.3 ± 3.3	6.9 ± 3.8	5.8 ± 2.7	6.1 ± 2.6	0.007
	MBR at unbuckle side (AU)	7.8 ± 2.4	7.8 ± 2.6	7.6 ± 2.4	6.9 ± 2.4	7.3 ± 2.5	0.017
	SFCT (µm)	237.3 ± 71.1	263.5 ± 83.5	240.1 ± 70.3	232.6 ± 55.9	232.1 ± 65.9	< 0.001
	Luminal area (mm^2^)	0.233 ± 0.068	0.256 ± 0.077	0.237 ± 0.071	0.227 ± 0.062	0.228 ± 0.058	< 0.001
	Stromal area (mm^2^)	0.123 ± 0.041	0.131 ± 0.039	0.122 ± 0.045	0.118 ± 0.031	0.118 ± 0.035	< 0.001
	IOP (mmHg)	13.1 ± 3.3	12.8 ± 2.6	13.9 ± 3.1	15.3 ± 3.5	15.0 ± 3.5	< 0.001
	MOPP (mmHg)	50.3 ± 9.1	47.5 ± 8.3	47.4 ± 8.8	49.9 ± 9.9	46.1 ± 10.4	0.174
	Flare	9.7 ± 24.1	12.5 ± 27.3	10.7 ± 16.3	6.1 ± 2.1	5.3 ± 2.2	0.708
Fellow eye	MBR (AU)	9.6 ± 3.5	9.5 ± 3.8	9.0 ± 2.8	9.1 ± 3.0	9.0 ± 2.6	0.380
	MBR at other area (AU)	6.7 ± 2.3	7.0 ± 2.5	6.6 ± 2.6	6.9 ± 2.2	7.0 ± 1.7	0.782
	SFCT (µm)	240.8 ± 72.1	239.1 ± 78.7	241.9 ± 74.0	238.4 ± 49.0	240.0 ± 78.5	0.454
	Luminal area (mm^2^)	0.243 ± 0.078	0.241 ± 0.079	0.246 ± 0.077	0.244 ± 0.065	0.241 ± 0.074	0.675
	Stromal area (mm^2^)	0.115 ± 0.034	0.115 ± 0.040	0.118 ± 0.043	0.113 ± 0.032	0.112 ± 0.033	0.466
	IOP (mmHg)	14.4 ± 3.4	14.0 ± 2.8	14.7 ± 4.4	14.3 ± 3.4	13.8 ± 2.6	0.446
	MOPP (mmHg)	49.8 ± 9.8	47.4 ± 10.2	48.3 ± 10.2	51.0 ± 10.7	47.3 ± 9.9	0.523
	Flare	5.5 ± 10.8	4.4 ± 2.5	4.6 ± 2.6	4.9 ± 2.2	4.8 ± 2.7	0.912

RRD: rhegmatogenous retinal detachment, MBR: mean blur rate, SFCT: subfoveal choroidal thickness, IOP: intraocular pressure, MOPP: mean ocular perfusion pressure.

In the eyes affected by RRD, the mean choroidal MBR at the buckle side was 7.4 ± 4.0 AU before surgery, 7.3 ± 3.3 AU at 1 week, 6.9 ± 3.8 AU at 4 weeks, 5.8 ± 2.7 AU at 8 weeks and 6.1 ± 2.6 AU at 12 weeks following surgery, and that at the unbuckle side was 7.8 ± 2.4 AU before surgery, 7.8 ± 2.6 AU at 1 week, 7.6 ± 2.4 AU at 4 weeks, 6.9 ± 2.4 AU at 8 weeks and 7.3 ± 2.5 AU at 12 weeks following surgery. There was a significant reduction in choroidal MBR at the buckle and unbuckle sides at 8 weeks following surgery compared with that before surgery in the affected eyes (P = 0.007 and P = 0.017, respectively).

The change in the choroidal MBR was not significantly correlated with the preoperative parameters of the refractive error, axial length, extent of quadrant-wise buckle (degrees), and extent of retinal detachment.

### Morphologic changes in choroid

**Figure 2:**
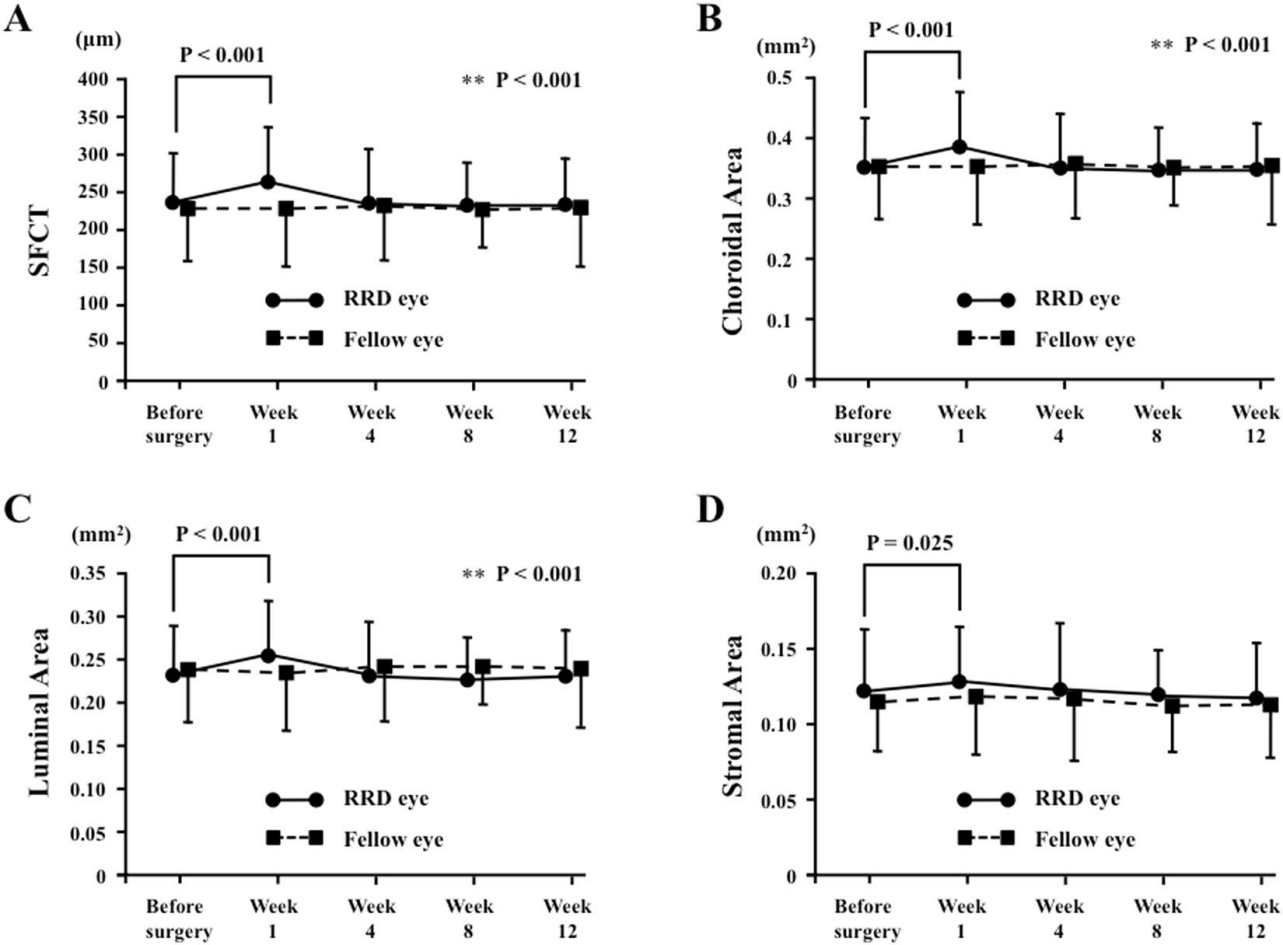
Horizontal SD-OCT images of an eye with macula-on RRD preoperatively and 1, 4 and 12 weeks postoperatively (**A**). The area of interest of the choroid is demarcated (**B**). The EDI-OCT image is converted to a binary image using ImageJ software (**C**). The rectangle surrounded by the red line was excised, and the dark areas were traced by the modified Niblack method. In the merged binarized images and the margins of traced areas, the light pixels were defined as the stromal, and the dark pixels as the luminal areas. SFCT at Week 1 was thicker than that preoperatively, and at weeks 4 and 12 postoperatively. The trend of SFCT with time coincided with that of the luminal area.

The mean SFCT in the operated eyes was 237.3 ± 71.1 µm before surgery, 263.5 ± 83.5 µm at 1 week, 240.1 ± 70.3 µm at 4 weeks, 232.6 ± 55.9 µm at 8 weeks and 232.1 ± 65.9 µm at 12 weeks following surgery (Fig. 2A). The mean SFCT at 1 week after surgery was significantly thicker than that before surgery (P < 0.001), while there was no significant difference between the SFCT preoperatively and after postoperative week 4. There was no significant change in SFCT over time in the fellow eyes.

**Figure 3:**
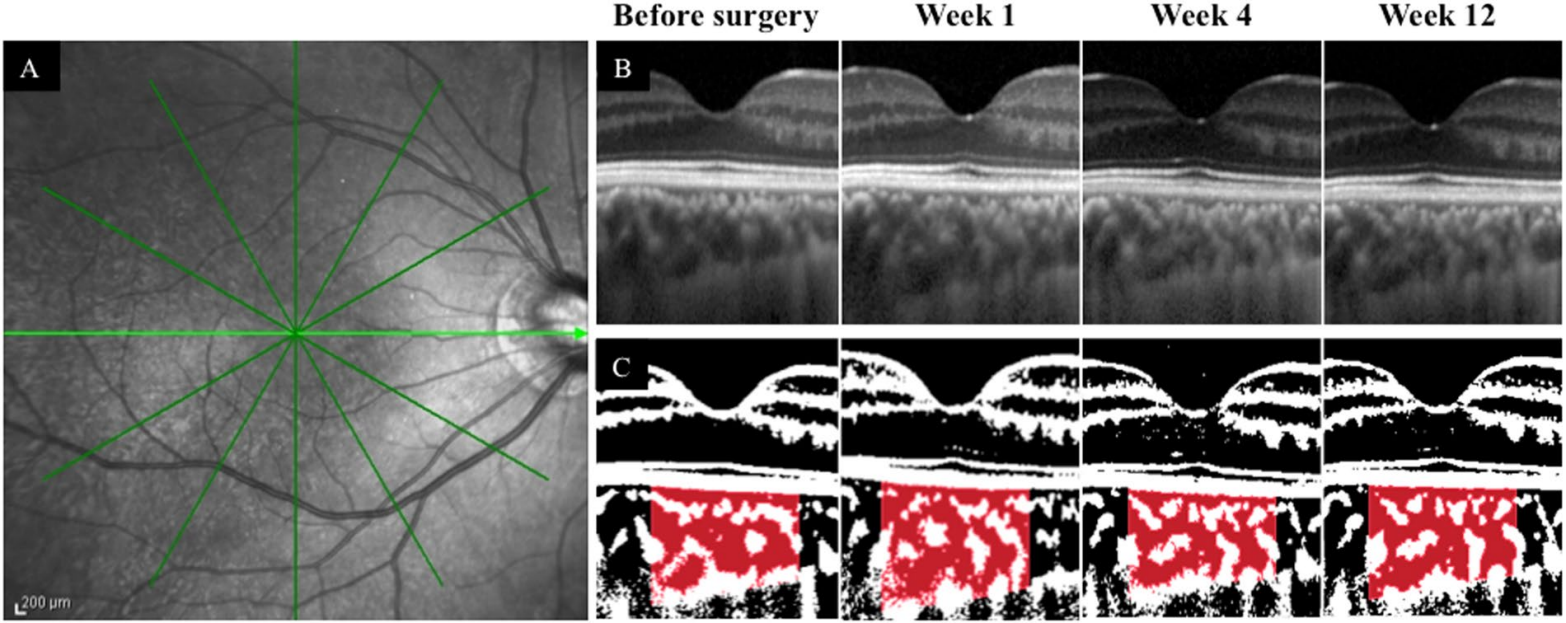
The mean subfoveal choroidal thickness (SFCT) at 1 week postoperatively was significantly thicker than that preoperatively in the operated eye (P < 0.001), while there was no significant change in the fellow eye throughout the follow-up period (**A**). The choroidal area (**B**), the luminal area (**C**) and the stromal area (**D**) at 1 week postoperatively were significantly increased compared with those preoperatively in the operated eye (P < 0.001, P < 0.001, P = 0.025, respectively), while there was no significant change in the fellow eye throughout the follow-up period.

The mean choroidal, luminal and stromal areas were 0.360 ± 0.101 mm^2^, 0.233 ± 0.068 mm^2^ and 0.123 ± 0.041 mm^2^, respectively (Fig. 2). These significantly increased to 0.387 ± 0.111 mm^2^, 0.256 ± 0.077 mm^2^, 0.131 ± 0.039 mm^2^, respectively, at one week following surgery (P < 0.001, P < 0.001, P = 0.025, respectively) (Figs 2 and 3), while there was no significant difference in those areas in the fellow eyes throughout the follow-up period.

SFCT significantly correlated with the choroidal, luminal, and stromal areas in the entire observation period (Table 3). The ratio of postoperative SFCT to preoperative SFCT correlated with that of the luminal area throughout the postoperative period but did not correlate with that of the stromal area throughout this period (Table 3).

**Table 3:** Result of Spearman’s rank correlation coefficient between the SFCT and variables determined by binarization method.

Parameter	Choroidal area	Luminal area	Stromal area
SFCT			
Before surgery	0.868^a^	0.901^a^	0.641^a^
Week 1	0.901^a^	0.908^a^	0.758^a^
Week 4	0.900^a^	0.881^a^	0.846^a^
Week 8	0.900^a^	0.865^a^	0.732^a^
Week 12	0.872^a^	0.877^a^	0.733^a^
Week 1/before surgery	0.258	0.423^c^	−0.085
Week 4/before surgery	0.596^b^	0.582^b^	0.049
Week 8/before surgery	0.730^a^	0.712^a^	0.231
Week 12/before surgery	0.578^b^	0.664^b^	0.105

SFCT = subfoveal choroidal thickness.

^a^ *p* < 0.001, ^b^ *p* < 0.01, ^c^ *p* < 0.05.

### Correlation between SFCT and other parameters

Regarding the correlation of variation of SFCT with that of other factors, the trend of SFCT coincided with that of the choroidal and luminal areas, IOP and macular choroidal MBR (P < 0.001, P < 0.001, P = 0.037, P = 0.039, respectively).

Multiple regression analysis in the ratio of SFCT and variable parameters at 1 week postoperatively to that preoperatively shows that only the ratio of SFCT was positively correlated with that of the luminal area (P = 0.018).

## Discussion

Our results showed that macular choroidal blood flow determined by LSFG did not change, while the choroidal blood flow at the buckle and unbuckle sides was reduced at 8 weeks following segmental scleral buckling with cryopexy procedures in the operated eye. The SFCT and the luminal area determined by the binarization method increased temporarily 1 week postoperatively. The ratio of postoperative SFCT to preoperative SFCT correlated with the luminal area but did not correlate with the stromal area throughout the postoperative period. In addition, the trend of the SFCT coincided with that of the luminal area postoperatively, while there was no morphologic change over time in the fellow eye. Multiple regression analysis demonstrates the SFCT at 1 week postoperatively:preoperatively was positively correlated with that of the luminal area.

Many reports have described ocular blood flow reduction following scleral bucking, generally including the encircling procedure–the element generally agreed to affect ocular blood flow. While some reports showed a progressive reduction and no return to the baseline value in choroidal blood flow after the buckling procedure^18^, others found a return in choroidal blood flow 3 to 6 months postoperatively^8, 10^. All these reports–some controversial–suggested that compression on the peripheral vasculature by indentation caused by the encircling buckle caused the reduction in ocular blood flow. The discrepancy among reports is partly due to the dependence of blood flow rate reduction upon the degree of constriction of the encircling band. However, in the present study, there was no significant reduction in choroidal MBR at the macula postoperatively.

We contrastingly performed segmental (not encircling) scleral buckling with an average of 92 degrees in all cases. Our objectives, follow-up period and measuring method were different from those of other reports, precluding direct comparison. However, our results indicated that partial compression force induced by segmental scleral buckling does not change choroidal blood flow at the macula. Ito *et al*. reported that scleral buckling procedures can cause subclinical disturbance of the choroidal circulation, even if encircling procedures are avoided. However, their exoplant silicone sponge was much wider (Mira No. 507, 509 in addition to No. 506), and the extent of quadrant-wise buckle, not mentioned. Our result indicates that relatively less invasive procedure of segmental (without encircling) scleral buckling may not affect macular blood flow.

There have been few reports describing the change in SFCT following scleral buckling. Kimura *et al*. reported a mean 13% temporary thickening in SFCT following segmental scleral buckling, and no significant difference after postoperative month 3^16^. Our result, with a temporary 10% thickening in SFCT, only at 1 week after segmental scleral buckling, demonstrates that our procedure causes less SFCT thickening than Kimura’s^16^, possibly due to the shorter extent of our quadrant-wise buckle (degrees) compared to Kimura’s average of 107 degrees. On the other hand, encircling reportedly causes greater thickening of SFCT, even in late postoperative phases^19^.

It has been reported that the percentage increase in SFCT is correlated with the percentage change in IOP after trabeculectomy^20^. This would indicate that changes in IOP can affect SFCT. However, in the present study, IOP marginally decreased by 2.3% at 1 week after surgery, although SFCT transiently increased by 10%. These results suggest that the decrease in IOP probably does not play the main role in the temporary thickening in SFCT at 1 week after surgery.

The exact mechanism by which scleral buckling affects SFCT remains unclear, but, the postoperative trend of SFCT determined by binarization coincided with that of the luminal area in our study, and multiple regression analysis shows that SFCT at 1 week postoperatively: preoperatively ratio was positively correlated only with that of the luminal area. Temporary alteration of the choroidal vessel area affects mainly the thickening of SFCT. Venous drainage obstruction induced by compression force of the scleral buckle apparently leads to the temporary thickening of SFCT in the acute postoperative phase, which would be related to compensation of the hemostasis in ocular circulation.

The stromal area was also temporally increased 1 week after segmental buckling in our binarization method, but changes did not coincide with those for SFCT. Histologically, the choroid comprises blood vessels and stromal tissues–the latter including pigment cells, smooth muscles, neurons, vascular walls, inflammatory cells and connective tissue–in which differentiation by binarization can increase inflammatory retinochoroidal disease^21^. Therefore, the temporary increase in stromal area indicates that ocular inflammation might contribute to the thickening of SFCT. One report attributes the temporary increase in SFCT to periocular inflammation caused by cryotherapy^16, 19^.

We know of no reports describing the relation between macular choroidal blood flow and morphological change following RRD surgery. Our results show that macular choroidal blood flow does not change and is not correlated with choroidal morphological change following segmental scleral buckling, perhaps because neither the increment of SFCT nor the luminal area is extensive, and venous drainage obstruction at the macula is exiguous.

In contrast to the macula, the choroidal blood flow in the buckle side and unbuckle side was significantly reduced at the late postoperative period of 8 weeks following scleral buckling surgery. Using the pulsatile ocular blood flow, Yokota *et al*. reported that the choroidal blood flow decreases after scleral buckling; this can reflect the whole choroidal blood flow^8^. It is not possible to accurately measure blood flow in areas with retinal detachment using LSFG. Accordingly, we measured blood flow at the buckle side in area without retinal detachment, which results that the area is not close to the area with the buckling element in the most of the present cases.

The compression force caused by segmental buckling on the peripheral vasculature probably does not increase with time postoperatively. In addition, as a treatment for RRD, significant changes in choroidal blood flow have also been reported following vitrectomy^22^. Accordingly, alternative mechanisms to compression forces would cause the late appearance of the reduction of the choroidal blood flow except for the macula. Trans-scleral cryopexy leads a devoid of choriocapillaris and choroidal large vessel and loss of the photoreceptor, which has the potential to cause a reduction in choroidal blood flow. The late appearance of the reduction in the choroidal blood flow at the buckle and unbuckle sides would be caused by cryopexy rather than the mechanical compression force from segmental buckling. The choroidal blood flow close to the buckling element should decrease because the choroid undergoing cryopexy gets atrophied with time. These results suggest that the entire choroidal blood flow would be reduced after buckling surgery because of the reduction of blood flow to some areas, except for the macula.

Macular choroidal blood flow is enriched to supply nutrition to the dense macular photoreceptor area for survival. In addition, choroidal thickness in the macular region is higher than in other areas. Taken together, the partial compression force induced by a relatively less invasive procedure of segmental (not encircling) scleral buckling may not affect macular blood flow, differing from other choroidal areas.

Limitations of this study included small sample size and short-term follow-up. Diurnal variations in choroidal thickness^23, 24^ and blood flow^25^ reportedly occur; similar diurnal patterns were reported on different days in SFCT^24^. We performed all examinations at approximately 12 PM to avoid diurnal variations, and SFCT in the fellow eyes showed no significant differences preoperatively and postoperatively, suggesting that diurnal fluctuations had little influence in our study. In addition, the first postoperative examination was at 1 week in our study, and SFCT may have been thicker at an earlier postoperative period than postoperative 1 week. Further studies with a larger number of patients and a wide range of postoperative periods are needed.

Our study indicates that venous drainage obstruction induced by partial compression force of scleral buckling leads to the thickening of SFCT in the acute postoperative phase. Although these results require careful interpretation since previous investigations used different parameters and measurement methods. We conclude that macular choroidal blood flow might be less susceptible to partial compression force because the blood flow at the macula, in contrast to the other areas, does not change following segmental scleral buckling.

## Methods

### Ethics statement

In this retrospective, cross-sectional single-centre study, the procedures used were approved by the Ethics Committee of the Nagoya University Hospital (Nagoya, Japan). The study conformed to the tenets of the Declaration of Helsinki.

### Subjects

We reviewed the medical records of all patients who had undergone segmental scleral buckling procedures at the Nagoya University Hospital from July 2013 to March 2015. All patients signed an informed consent form before surgery.

All patients underwent a comprehensive ophthalmic examination including the measurement of intraocular pressure (IOP) and axial length, slit-lamp examination, fundus examination and OCT preoperatively and 1,4, 8 and 12 weeks postoperatively.

All patients were asked to abstain from alcoholic and caffeinated beverages on the morning of the day of the examination. The pupil was dilated 30 minutes before the examinations. The subjects rested for 10 to 15 min in a quiet dark room before the examination. All examinations were performed in the sitting position at approximately 12 PM to avoid diurnal variations^16, 23, 25^. The axial lengths were measured with partial optical coherence interferometry (IOLMaster; Carl Zeiss Meditec, La Jolla, CA). The IOP was measured with a handheld tonometer (Icare; TiolatOy, Helsinki, Finland). The systolic blood pressure (SBP) and the diastolic blood pressure (DBP) were measured with an automatic sphygmomanometer (CH-483C; Citizen, Tokyo, Japan). The mean arterial blood pressure (MAP) and mean ocular perfusion pressure (MOPP) were calculated as follows: MAP = DBP + 1/3(SBP-DBP), MOPP = 2/3MAP − IOP.

### LSFG

The LSFG-NAVI was used to determine choroidal blood flow. The principles of LSFG have been described in detail^26^. To evaluate macular choroidal blood flow, the centre of a rectangle (250 × 250 pixels, degree: 6.31° × 6.31°) was placed at the fovea (Fig. 1A). To evaluate choroidal blood flow at the buckle and unbuckle sides, a rectangle (60 × 60 pixels, degree: 1.5° × 1.5°) was placed at the area where visible retinal vessels were not observed (Fig. 1B). The LSFG was measured three times at each time-point in all eyes, and the averages of the variables were calculated. The area same as the operated eye in the fellow eye was used as the control.

### Measurement of choroidal thickness and differentiation of luminal and stromal areas

Choroidal images were obtained by SD-OCT (Spectralis OCT, Heidelberg Engineering, Heidelberg, Germany). The choroidal thickness was measured with SD-OCT using the enhanced depth-imaging (EDI) technique. The SFCT was measured as the distance from the hyper-reflective RPE line to the choroid–sclera border with the calliper tool on SD-OCT.

Binarization of the choroidal area in the EDI-OCT image was performed by the modified Niblack’s method^17^. The EDI-OCT image was analysed using ImageJ software (ImageJ version 1.47, NIH, Bethesda, MD). The examined area was 1,500 μm wide in the subfoveal choroid, extending vertically from the RPE to the chorioscleral border (Fig. 3). The light pixels were defined as the interstitial areas, and the dark pixels were defined as the luminal areas. After adding the data of the distance of each pixel, the luminal and interstitial areas were automatically calculated. Two clinicians –masked to the other findings–measured the area.

### Surgical technique

In all patients, retinal breaks were identified and treated by transscleral cryotherapy. Mattress sutures were placed 7.0 to 7.5 mm apart with 4–0 supramid (Kono, Chiba, Japan) for the circumferential segmental buckle and a silicone sponge (Mira No. 506; Mira, Inc, Waltham, MA) was sutured as an explant in all cases. Scleral dissection, extraocular muscle disinsertion and concomitant encircling were not required for any patients. Subretinal fluid drainage was performed if necessary. Intraocular tamponade was not used for all cases. Dexamethasone (MSD K.K., Tokyo, Japan) was injected subconjunctivally at the end of surgery.

No intraoperative complication was encountered. Reattachment of the retina was achieved in all patients in the initial surgery.

## Exclusion Criteria

The exclusion criteria included presence of any optic disc abnormalities such as glaucoma or optic disc atrophy, history of intraocular surgery, presence of vitreous haemorrhage, severe cataract and medical conditions that could influence the haemodynamics of the eye, such as diabetes, hypertension, arrhythmia and vascular diseases.

## Statistical analyses

We evaluated the changes in choroidal MBR, SFCT, choroidal area, luminal area, stromal area and IOP, MOPP and flare and the correlation of variation of SFCT with that of other factors from five fixed time points (before and 1, 4, 8 and 12-week(s) after surgery). The strength of the mixed model for analysis of dynamic, longitudinal data is that it can describe each individual’s pattern of change even in the face of missing data points^27^. Therefore, we used a mixed model to incorporate the appropriate covariates between repeated measured values over time.

Specifically, we assumed the following model:

$${{\rm{y}}}_{{\rm{ij}}}={a}_{i}+f({t}_{j},{G}_{i}\,:b)+{{\rm{\varepsilon }}}_{{\rm{ij}}}$$

i(subject)=1, …, 46, j(time) = 0, 1, 4, 8, 12 where yij is the variables at time j on subject i; ${a}_{i}$ is a subject-specific random effect; *Gi* = 1 indicates affected eye and 0 indicates unaffected eye. The function f(*tj:b*) represents a fixed effect of time on the variables. For the residual term e_ij_ of the values, we assumed a heterogeneous compound symmetry structure within patients.

All statistical analyses were performed with SAS9.4 (SAS Inc., Cary). A P value < 0.05 was considered statistically significant.
